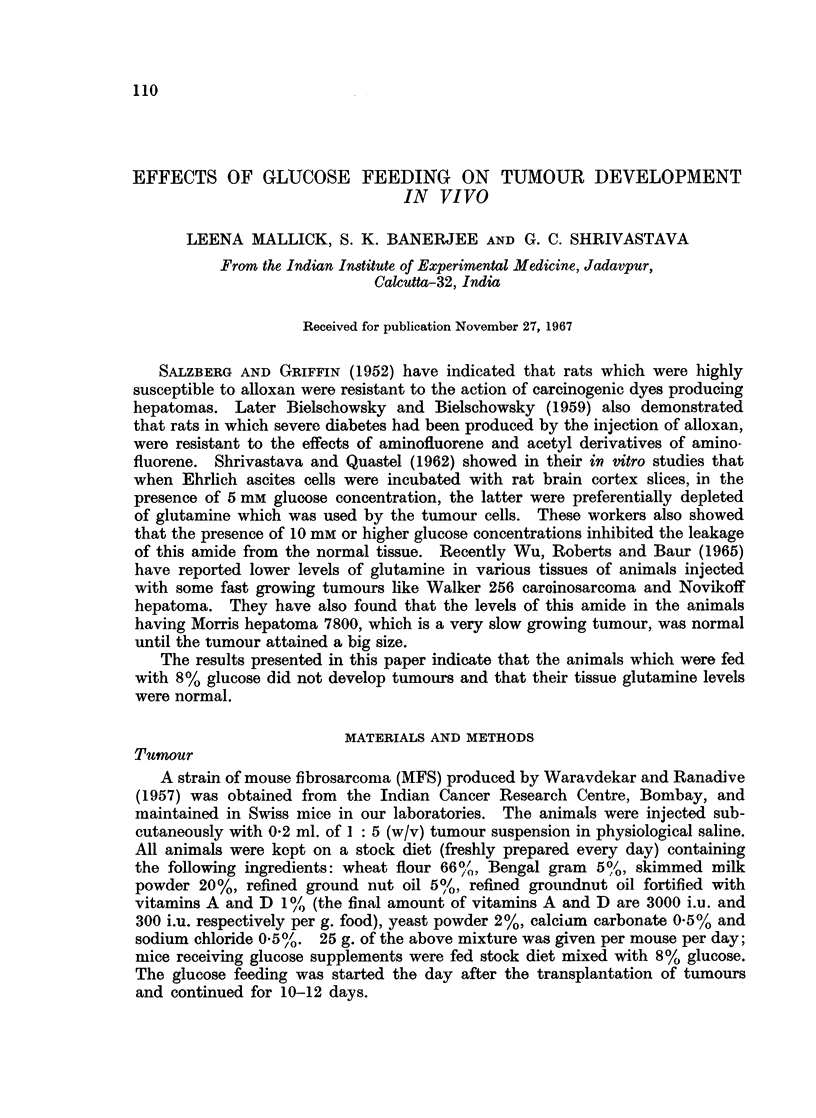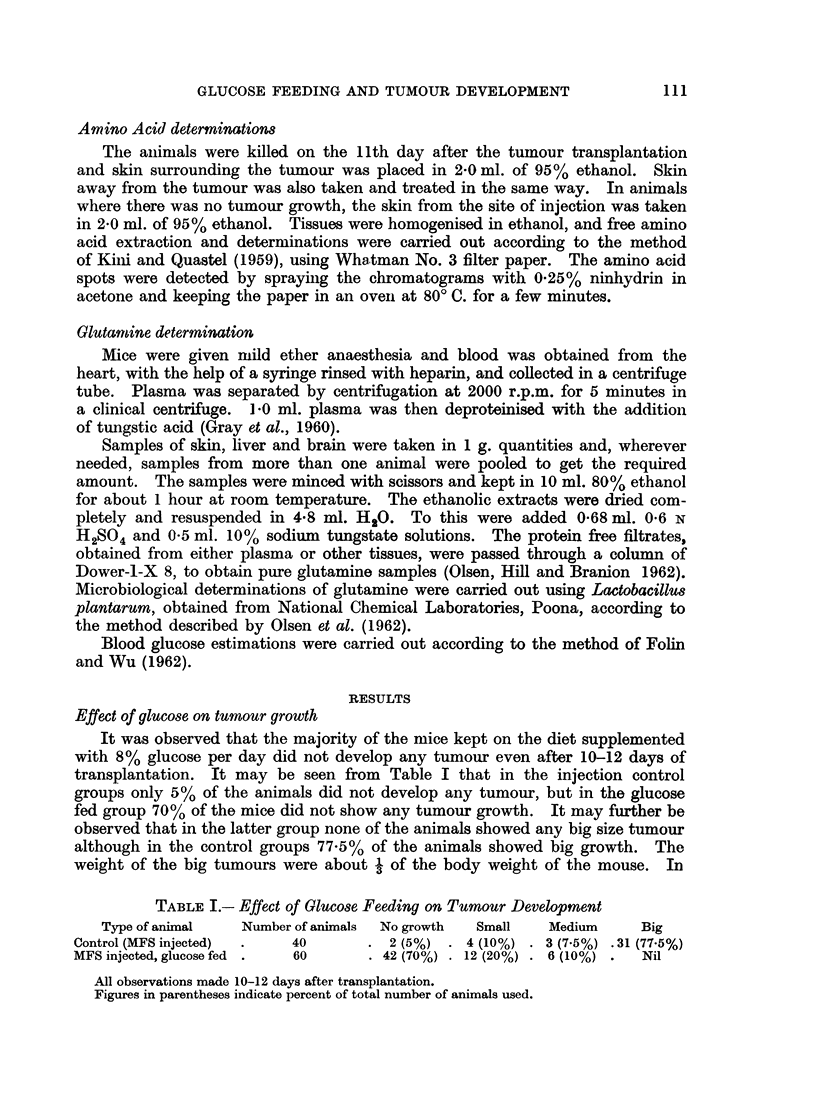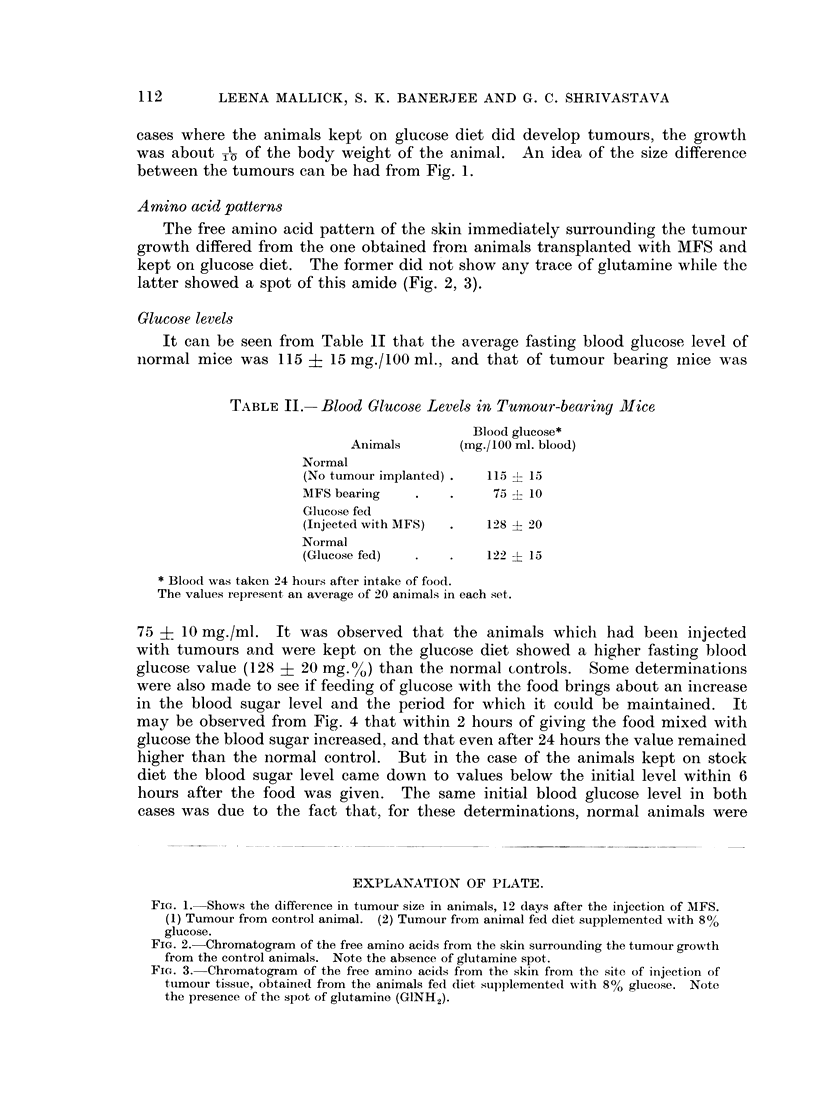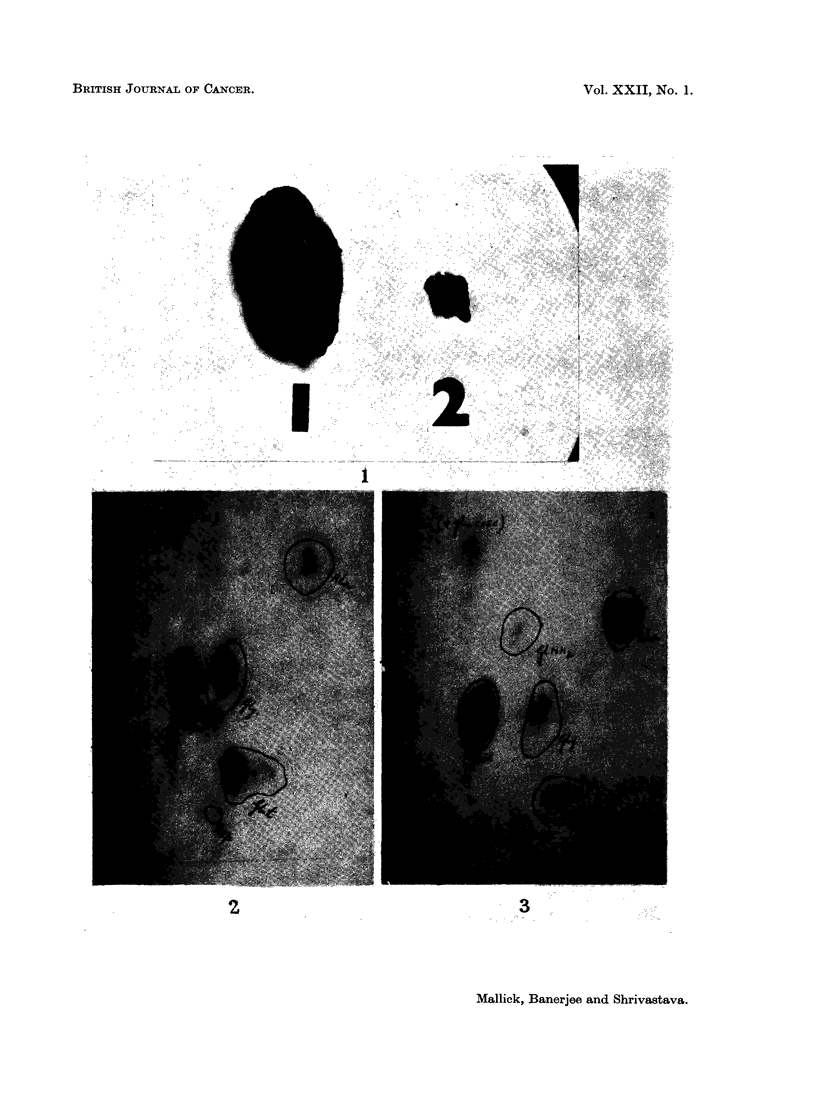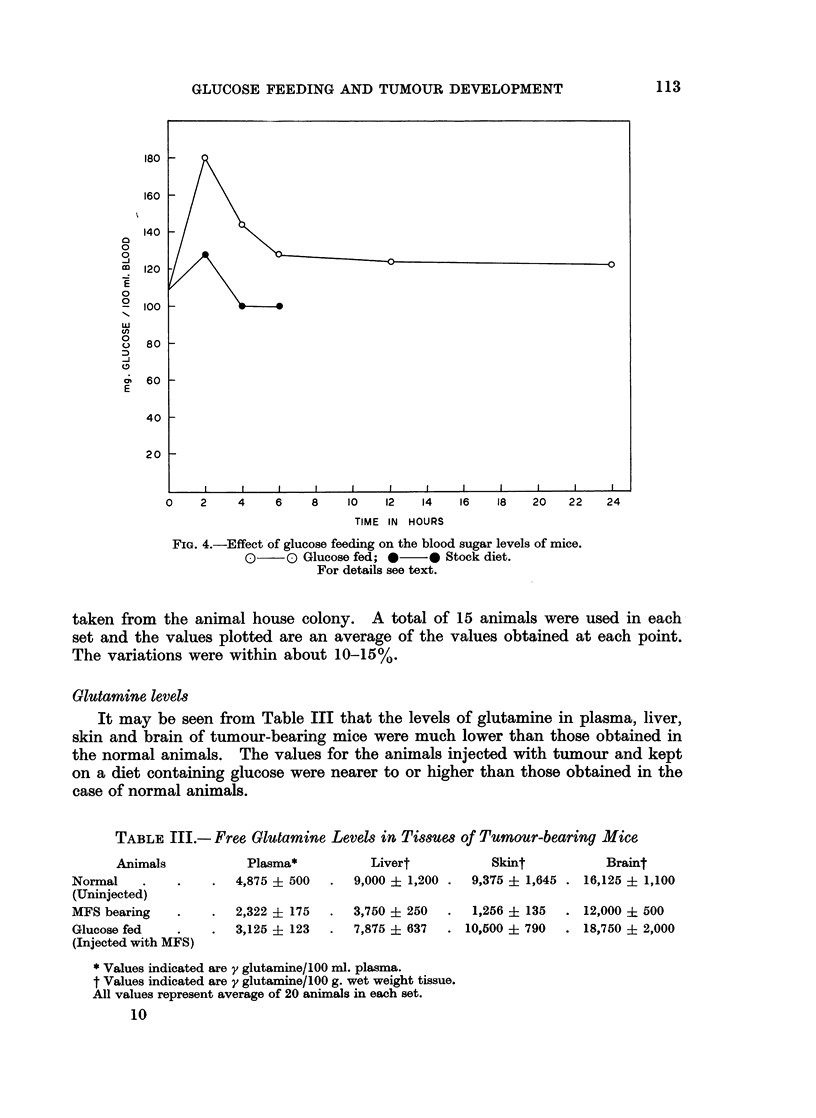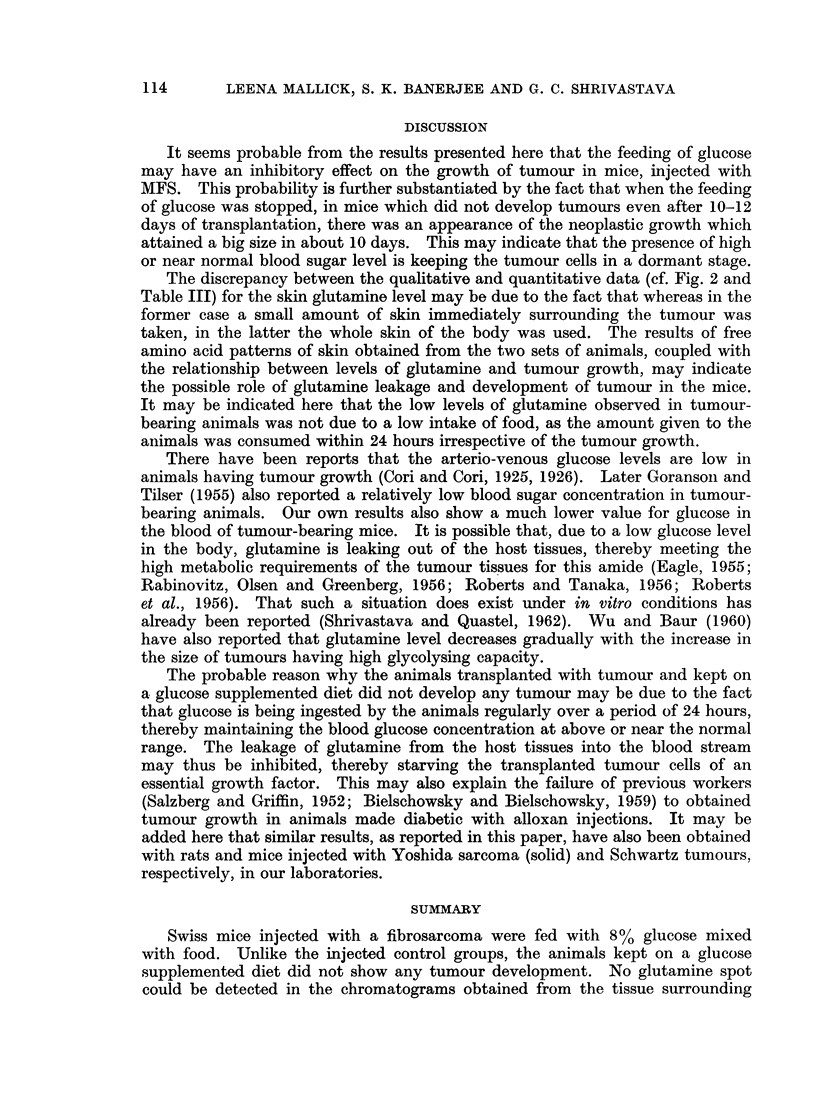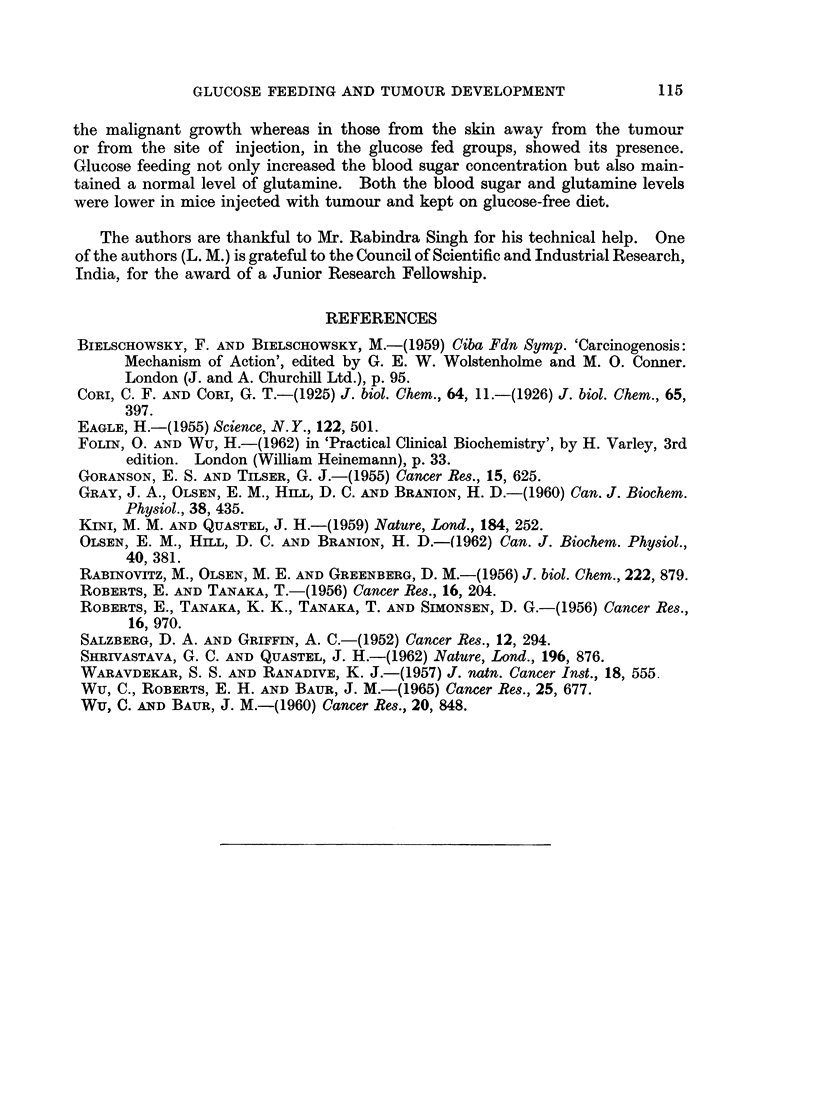# Effects of glucose feeding on tumour development in vivo.

**DOI:** 10.1038/bjc.1968.15

**Published:** 1968-03

**Authors:** L. Mallick, S. K. Banerjee, G. C. Shrivastava

## Abstract

**Images:**


### EFFECTS OF GLUCOSE FEEDING ON TUMOUR DEVELOPMENT *IN VIVO*

LEENA MALLICK, S. K. BANERJEE AND G. C. SHRIVASTAVA

*From the Indian Institute of Experimental Medicine, Jadavpur, Calcutta-32, India*

Received for publication November 27, 1967

SALZBERG AND GRIFFIN (1952) have indicated that rats which were highly susceptible to alloxan were resistant to the action of carcinogenic dyes producing hepatomas. Later Bielschowsky and Bielschowsky (1959) also demonstrated that rats in which severe diabetes had been produced by the injection of alloxan, were resistant to the effects of aminofluorene and acetyl derivatives of aminofluorene. Shrivastava and Quastel (1962) showed in their *in vitro* studies that when Ehrlich ascites cells were incubated with rat brain cortex slices, in the presence of 5 mM glucose concentration, the latter were preferentially depleted of glutamine which was used by the tumour cells. These workers also showed that the presence of 10 mM or higher glucose concentrations inhibited the leakage of this amide from the normal tissue. Recently Wu, Roberts and Baur (1965) have reported lower levels of glutamine in various tissues of animals injected with some fast growing tumours like Walker 256 carcinosarcoma and Novikoff hepatoma. They have also found that the levels of this amide in the animals having Morris hepatoma 7800, which is a very slow growing tumour, was normal until the tumour attained a big size.

The results presented in this paper indicate that the animals which were fed with 8% glucose did not develop tumours and that their tissue glutamine levels were normal.

### MATERIALS AND METHODS

### *Tumour*

A strain of mouse fibrosarcoma (MFS) produced by Waravdekar and Ranadive (1957) was obtained from the Indian Cancer Research Centre, Bombay, and maintained in Swiss mice in our laboratories. The animals were injected subcutaneously with 0·2 ml. of 1 : 5 (w/v) tumour suspension in physiological saline. All animals were kept on a stock diet (freshly prepared every day) containing the following ingredients: wheat flour 66%, Bengal gram 5%, skimmed milk powder 20%, refined ground nut oil 5%, refined groundnut oil fortified with vitamins A and D 1% (the final amount of vitamins A and D are 3000 i.u. and 300 i.u. respectively per g. food), yeast powder 2%, calcium carbonate 0·5% and sodium chloride 0·5%. 25 g. of the above mixture was given per mouse per day; mice receiving glucose supplements were fed stock diet mixed with 8% glucose. The glucose feeding was started the day after the transplantation of tumours and continued for 10–12 days.

*Amino Acid determinations*

The animals were killed on the 11th day after the tumour transplantation and skin surrounding the tumour was placed in 2·0 ml. of 95% ethanol. Skin away from the tumour was also taken and treated in the same way. In animals where there was no tumour growth, the skin from the site of injection was taken in 2·0 ml. of 95% ethanol. Tissues were homogenised in ethanol, and free amino acid extraction and determinations were carried out according to the method of Kini and Quastel (1959), using Whatman No. 3 filter paper. The amino acid spots were detected by spraying the chromatograms with 0·25% ninhydrin in acetone and keeping the paper in an oven at 80° C. for a few minutes.

*Glutamine determination*

Mice were given mild ether anaesthesia and blood was obtained from the heart, with the help of a syringe rinsed with heparin, and collected in a centrifuge tube. Plasma was separated by centrifugation at 2000 r.p.m. for 5 minutes in a clinical centrifuge. 1·0 ml. plasma was then deproteinised with the addition of tungstic acid (Gray *et al.*, 1960).

Samples of skin, liver and brain were taken in 1 g. quantities and, wherever needed, samples from more than one animal were pooled to get the required amount. The samples were minced with scissors and kept in 10 ml. 80% ethanol for about 1 hour at room temperature. The ethanolic extracts were dried completely and resuspended in 4·8 ml. $H_2O$. To this were added 0·68 ml. 0·6 N $H_2SO_4$ and 0·5 ml. 10% sodium tungstate solutions. The protein free filtrates, obtained from either plasma or other tissues, were passed through a column of Dower-1-X 8, to obtain pure glutamine samples (Olsen, Hill and Branion 1962). Microbiological determinations of glutamine were carried out using *Lactobacillus plantarum*, obtained from National Chemical Laboratories, Poona, according to the method described by Olsen *et al.* (1962).

Blood glucose estimations were carried out according to the method of Folin and Wu (1962).

### RESULTS

*Effect of glucose on tumour growth*

It was observed that the majority of the mice kept on the diet supplemented with 8% glucose per day did not develop any tumour even after 10–12 days of transplantation. It may be seen from Table I that in the injection control groups only 5% of the animals did not develop any tumour, but in the glucose fed group 70% of the mice did not show any tumour growth. It may further be observed that in the latter group none of the animals showed any big size tumour although in the control groups 77·5% of the animals showed big growth. The weight of the big tumours were about ⅓ of the body weight of the mouse. In

TABLE I.—*Effect of Glucose Feeding on Tumour Development*

| Type of animal | Number of animals | No growth | Small | Medium | Big |
|---|---|---|---|---|---|
| Control (MFS injected) | 40 | 2 (5%) | 4 (10%) | 3 (7·5%) | 31 (77·5%) |
| MFS injected, glucose fed | 60 | 42 (70%) | 12 (20%) | 6 (10%) | Nil |

All observations made 10–12 days after transplantation.
Figures in parentheses indicate percent of total number of animals used.

cases where the animals kept on glucose diet did develop tumours, the growth was about $\frac{1}{10}$ of the body weight of the animal. An idea of the size difference between the tumours can be had from Fig. 1.

*Amino acid patterns*

The free amino acid pattern of the skin immediately surrounding the tumour growth differed from the one obtained from animals transplanted with MFS and kept on glucose diet. The former did not show any trace of glutamine while the latter showed a spot of this amide (Fig. 2, 3).

*Glucose levels*

It can be seen from Table II that the average fasting blood glucose level of normal mice was $115 \pm 15$ mg./100 ml., and that of tumour bearing mice was

TABLE II.—*Blood Glucose Levels in Tumour-bearing Mice*

| Animals | Blood glucose* (mg./100 ml. blood) |
|---|---|
| Normal (No tumour implanted) . | $115 \pm 15$ |
| MFS bearing . . | $75 \pm 10$ |
| Glucose fed (Injected with MFS) . | $128 \pm 20$ |
| Normal (Glucose fed) . . | $122 \pm 15$ |

* Blood was taken 24 hours after intake of food.
The values represent an average of 20 animals in each set.

$75 \pm 10$ mg./ml. It was observed that the animals which had been injected with tumours and were kept on the glucose diet showed a higher fasting blood glucose value ($128 \pm 20$ mg.%) than the normal controls. Some determinations were also made to see if feeding of glucose with the food brings about an increase in the blood sugar level and the period for which it could be maintained. It may be observed from Fig. 4 that within 2 hours of giving the food mixed with glucose the blood sugar increased, and that even after 24 hours the value remained higher than the normal control. But in the case of the animals kept on stock diet the blood sugar level came down to values below the initial level within 6 hours after the food was given. The same initial blood glucose level in both cases was due to the fact that, for these determinations, normal animals were

EXPLANATION OF PLATE.

FIG. 1.—Shows the difference in tumour size in animals, 12 days after the injection of MFS. (1) Tumour from control animal. (2) Tumour from animal fed diet supplemented with 8% glucose.

FIG. 2.—Chromatogram of the free amino acids from the skin surrounding the tumour growth from the control animals. Note the absence of glutamine spot.

FIG. 3.—Chromatogram of the free amino acids from the skin from the site of injection of tumour tissue, obtained from the animals fed diet supplemented with 8% glucose. Note the presence of the spot of glutamine ($GlNH_2$).

1

2

3

Mallick, Banerjee and Shrivastava.

FIG. 4.—Effect of glucose feeding on the blood sugar levels of mice.
○——○ Glucose fed; ●——● Stock diet.
For details see text.

taken from the animal house colony. A total of 15 animals were used in each set and the values plotted are an average of the values obtained at each point. The variations were within about 10–15%.

*Glutamine levels*

It may be seen from Table III that the levels of glutamine in plasma, liver, skin and brain of tumour-bearing mice were much lower than those obtained in the normal animals. The values for the animals injected with tumour and kept on a diet containing glucose were nearer to or higher than those obtained in the case of normal animals.

TABLE III.—*Free Glutamine Levels in Tissues of Tumour-bearing Mice*

| Animals | Plasma* | Liver† | Skin† | Brain† |
|---|---|---|---|---|
| Normal (Uninjected) | 4,875 ± 500 | 9,000 ± 1,200 | 9,375 ± 1,645 | 16,125 ± 1,100 |
| MFS bearing | 2,322 ± 175 | 3,750 ± 250 | 1,256 ± 135 | 12,000 ± 500 |
| Glucose fed (Injected with MFS) | 3,125 ± 123 | 7,875 ± 637 | 10,500 ± 790 | 18,750 ± 2,000 |

* Values indicated are γ glutamine/100 ml. plasma.
† Values indicated are γ glutamine/100 g. wet weight tissue.
All values represent average of 20 animals in each set.

### DISCUSSION

It seems probable from the results presented here that the feeding of glucose may have an inhibitory effect on the growth of tumour in mice, injected with MFS. This probability is further substantiated by the fact that when the feeding of glucose was stopped, in mice which did not develop tumours even after 10–12 days of transplantation, there was an appearance of the neoplastic growth which attained a big size in about 10 days. This may indicate that the presence of high or near normal blood sugar level is keeping the tumour cells in a dormant stage.

The discrepancy between the qualitative and quantitative data (cf. Fig. 2 and Table III) for the skin glutamine level may be due to the fact that whereas in the former case a small amount of skin immediately surrounding the tumour was taken, in the latter the whole skin of the body was used. The results of free amino acid patterns of skin obtained from the two sets of animals, coupled with the relationship between levels of glutamine and tumour growth, may indicate the possible role of glutamine leakage and development of tumour in the mice. It may be indicated here that the low levels of glutamine observed in tumour-bearing animals was not due to a low intake of food, as the amount given to the animals was consumed within 24 hours irrespective of the tumour growth.

There have been reports that the arterio-venous glucose levels are low in animals having tumour growth (Cori and Cori, 1925, 1926). Later Goranson and Tilser (1955) also reported a relatively low blood sugar concentration in tumour-bearing animals. Our own results also show a much lower value for glucose in the blood of tumour-bearing mice. It is possible that, due to a low glucose level in the body, glutamine is leaking out of the host tissues, thereby meeting the high metabolic requirements of the tumour tissues for this amide (Eagle, 1955; Rabinovitz, Olsen and Greenberg, 1956; Roberts and Tanaka, 1956; Roberts *et al.*, 1956). That such a situation does exist under *in vitro* conditions has already been reported (Shrivastava and Quastel, 1962). Wu and Baur (1960) have also reported that glutamine level decreases gradually with the increase in the size of tumours having high glycolysing capacity.

The probable reason why the animals transplanted with tumour and kept on a glucose supplemented diet did not develop any tumour may be due to the fact that glucose is being ingested by the animals regularly over a period of 24 hours, thereby maintaining the blood glucose concentration at above or near the normal range. The leakage of glutamine from the host tissues into the blood stream may thus be inhibited, thereby starving the transplanted tumour cells of an essential growth factor. This may also explain the failure of previous workers (Salzberg and Griffin, 1952; Bielschowsky and Bielschowsky, 1959) to obtained tumour growth in animals made diabetic with alloxan injections. It may be added here that similar results, as reported in this paper, have also been obtained with rats and mice injected with Yoshida sarcoma (solid) and Schwartz tumours, respectively, in our laboratories.

### SUMMARY

Swiss mice injected with a fibrosarcoma were fed with 8% glucose mixed with food. Unlike the injected control groups, the animals kept on a glucose supplemented diet did not show any tumour development. No glutamine spot could be detected in the chromatograms obtained from the tissue surrounding the malignant growth whereas in those from the skin away from the tumour or from the site of injection, in the glucose fed groups, showed its presence. Glucose feeding not only increased the blood sugar concentration but also maintained a normal level of glutamine. Both the blood sugar and glutamine levels were lower in mice injected with tumour and kept on glucose-free diet.

The authors are thankful to Mr. Rabindra Singh for his technical help. One of the authors (L. M.) is grateful to the Council of Scientific and Industrial Research, India, for the award of a Junior Research Fellowship.

### REFERENCES

Bielschowsky, F. and Bielschowsky, M.—(1959) *Ciba Fdn Symp.* 'Carcinogenosis: Mechanism of Action', edited by G. E. W. Wolstenholme and M. O. Conner. London (J. and A. Churchill Ltd.), p. 95.

Cori, C. F. and Cori, G. T.—(1925) *J. biol. Chem.*, **64**, 11.—(1926) *J. biol. Chem.*, **65**, 397.

Eagle, H.—(1955) *Science, N.Y.*, **122**, 501.

Folin, O. and Wu, H.—(1962) in 'Practical Clinical Biochemistry', by H. Varley, 3rd edition. London (William Heinemann), p. 33.

Goranson, E. S. and Tilser, G. J.—(1955) *Cancer Res.*, **15**, 625.

Gray, J. A., Olsen, E. M., Hill, D. C. and Branion, H. D.—(1960) *Can. J. Biochem. Physiol.*, **38**, 435.

Kini, M. M. and Quastel, J. H.—(1959) *Nature, Lond.*, **184**, 252.

Olsen, E. M., Hill, D. C. and Branion, H. D.—(1962) *Can. J. Biochem. Physiol.*, **40**, 381.

Rabinovitz, M., Olsen, M. E. and Greenberg, D. M.—(1956) *J. biol. Chem.*, **222**, 879.

Roberts, E. and Tanaka, T.—(1956) *Cancer Res.*, **16**, 204.

Roberts, E., Tanaka, K. K., Tanaka, T. and Simonsen, D. G.—(1956) *Cancer Res.*, **16**, 970.

Salzberg, D. A. and Griffin, A. C.—(1952) *Cancer Res.*, **12**, 294.

Shrivastava, G. C. and Quastel, J. H.—(1962) *Nature, Lond.*, **196**, 876.

Waravdekar, S. S. and Ranadive, K. J.—(1957) *J. natn. Cancer Inst.*, **18**, 555.

Wu, C., Roberts, E. H. and Baur, J. M.—(1965) *Cancer Res.*, **25**, 677.

Wu, C. and Baur, J. M.—(1960) *Cancer Res.*, **20**, 848.